# Editorial: The Janus Face of Language: Where Are the Emotions in Words and Where Are the Words in Emotions?

**DOI:** 10.3389/fpsyg.2018.00650

**Published:** 2018-05-17

**Authors:** Cornelia Herbert, Thomas Ethofer, Andreas J. Fallgatter, Peter Walla, Georg Northoff

**Affiliations:** ^1^Department of Applied Emotion and Motivation Research, Institute of Psychology and Education, University of Ulm, Ulm, Germany; ^2^Department of Psychiatry, University of Tübingen, Tübingen, Germany; ^3^Department for Biomedical Resonance, University of Tübingen, Tübingen, Germany; ^4^LEAD Graduate School, University of Tuebingen, Tuebingen, Germany; ^5^Cognitive Neuroscience & Behaviour Lab (CanBeLab), Department of Psychology, Webster Vienna Private University, Vienna, Austria; ^6^School of Psychology, University of Newcastle, Callaghan, NSW, Australia; ^7^Faculty of Psychology, Vienna University, Vienna, Austria; ^8^Mind, Brain Imaging and Neuroethics, University of Ottawa Institute of Mental Health Research, University of Ottawa, Ottawa, ON, Canada

**Keywords:** emotion and language, embodiment, neuronal and behavioral mechanisms of emotional word processing, language as context, mood, multilingualism, poetic aesthetics, self-reference

## Introduction: where are the emotions in words?

We text, blog, twitter and tweet, we write each other emails, poems and love letters. Ever since in human history, people have been using language to communicate emotions and feelings, well knowing that words can hurt or heal. Thus, considering everyday experiences, there is no doubt that written language constitutes a most powerful tool for inducing emotions in self and others—and for eliciting emotional responses in the sender and perceiver of a message even when no direct face to face communication is possible.

However, what happens so naturally and effortlessly in everyday life has become a subject of intensive scientific debate. Can language, specifically written language in terms of single words elicit emotions? And if so, where are the emotions in words and where are the words in emotions?

Theoretically, the answer to these questions is anything than trivial. Traditionally, language has been considered a purely cognitive function of the human mind; a property of the mind that evolved for the purpose of representing individual experiences in an abstract way, independent from sensory and motor experience and independent from bodily sensations including emotions (for a discussion see Chapter 1 in this book). In this view, reading emotion-related or emotional words such as “snake” or “fear” may activate the semantic meaning of the word including its emotional meaning; readers may even infer from reading that snakes are harmful and threatening creatures; nonetheless, this knowledge would be stored in a purely amodal fashion. As a result, readers would be unable to bodily and affectively feel what they are reading because the crucial link between mental states and sensory, motor and peripheral (bodily) changes characterizing emotions would be missing. In other words, viewed from a pure cognitive approach of language, emotions and their perceptual, sensory and motor consequences can be expressed linguistically. However, the linguistic description and semantic representation of an emotion will not be accompanied by physiological bodily changes or by affective experiences of arousal, or by bodily feelings of pleasure and displeasure nor by changes in motivational behavior of approach or avoidance.

In recent years there have been changes with respect to the understanding of mind-body interactions and the role language may play in emotion processing and emotion regulation. In the past 15 years, a number of studies have been conducted at the interface of language and emotion, most if not all of them accumulated empirical evidence against the theoretical belief of a purely cognitive-based foundation of language (e.g., see Chapter 1–4 in this book).

## Emotional word processing—core dimensions, time course and brain structures

Several studies investigated the neurophysiological and psychophysiological correlates of emotional word processing to determine whether the processing of emotions from words and the processing of emotions from pictures or faces share the same neurophysiological mechanisms (e.g., Kissler et al., 2006, 2007; Herbert et al., 2008; Citron, 2012; Mavratzakis et al., 2016; see Bayer and Schacht; Palazova in this book for an overview). Methodologically, most studies used high-density electroencephalography (EEG) or functional magnetic resonance imaging (FMRI) techniques either alone or in combination with behavioral and subjective self-report measures.

Investigating emotional word processing in the brain showed that reading emotional words vs. neutral words increases neural activity in the ventral visual processing stream (involved in object recognition) within the first 200 ms after word presentation; i.e., in the same early time-windows reported in EEG studies investigating emotional picture processing (e.g., Junghöfer et al., 2001; Kissler et al., 2007; Herbert et al., 2008; see Bayer and Schacht, in this book for an overview). For words, occasionally, even earlier emotional facilitation effects have been reported indicating that emotional content is able to circumvent in-depth semantic analysis (e.g., Kissler and Herbert, 2013; see Palazova in this book for discussion). Interestingly, results from imaging studies suggested that these stimulus-driven neural activity changes are likely to be caused by reentrant processing between the amygdala and the ventral visual processing system (e.g., Herbert et al., 2009; see Flaisch et al.; Eden et al. in this book for a discussion). Furthermore, under some instances, processing of emotional words may also lead to changes in approach and avoidance behavior (e.g., Herbert and Kissler, 2010) and to specific approach- and avoidance related behavioral response patterns (see Citron et al. for an overview in this book).

Taken together, the results do not support the idea that language representations in the brain are cut off from perception, actions and emotions. Instead the results argue in favor of a common coding principle of how the brain represents and processes emotional information—be this information abstract or concrete, verbal or non-verbal. Emotional stimuli may therefore—regardless of the stimulus type (pictures, faces or words)—elicit changes in central autonomic arousal and in specific appraisals related to pleasure and displeasure. Nevertheless, as suggested by functional imaging and EEG source analysis studies, the activation of brain structures involved in the top-down regulation of visual attention can significantly differ during processing of emotional pictures vs. emotional words; also when words are used as task-related distracters vs. targets (e.g., see Flaisch et al.; Hinojosa et al. in this book).

This raises questions about whether the emotional content of a word is embodied during reading: i.e., can readers affectively experience and feel what they are reading? And if so, when during reading does this kind of embodied processing occur? Undeniably, many if not all languages are rich of emotional words, suggesting a tight connection between written words and felt emotions. Previous research exploring the structure of affective ratings in large emotional word corpora and different languages suggested two dimensional emotional factors of valence (positive vs. negative) and arousal (being physiologically calm vs. aroused). These two factors seem to explain most of the variance of the affective ratings of words (e.g., see Jacobs et al. in this book for an overview). More recent studies found that other stimulus appraisal factors related to sensing, acting and feeling may also play a role (e.g., see Imbir et al.; Jacobs et al. in this book for an overview). All in all, this suggests on the one hand, fast and non-reflective appraisal of words according to the bodily arousal of the words and on the other hand, temporally slower and reflective evaluation of the word's personal self-, motivational or emotional relevance.

The distinction between pre-reflective, arousal-driven vs. reflective and valence-driven appraisal checks is well in line with what EEG studies investigating the time course of emotional word processing during passive reading, lexical decision or rapid serial visual presentation suggested (Herbert et al., 2006, 2008; Kissler et al., 2007, 2009; Carretié et al., 2008; Schacht and Sommer, 2009; Hinojosa et al., 2010): a rapid and selective processing of highly arousing emotional words of positive and negative valence in the time window of, for instance, the early posterior negativity (EPN) and a temporally later in-depth semantic processing of emotional words according to their emotional valence (positive vs. negative) in, for instance, the time windows of the N400 and LPP (e.g., Herbert et al., 2006, 2008; Kissler et al., 2009; see Palazova; Bayer and Schacht, in this book for a discussion). Therefore, the emotional significance of a word may be quickly appraised according to its physiological arousal and its emotional intensity. However, at these early bottom-up driven stages of emotional word processing the subjective feelings that arise from this processing may at this stage of word processing not be consciously, conceptually and semantically available for the reader although they arise from verbal input (Herbert, 2015; see e.g., Lindquist; Ensie Abbassi et al. in this book for a theoretical discussion). Subjective feelings may be consciously, conceptually and semantically available for the reader only during later stages of word processing.

## Effects of mood, intrapersonal and sublexical factors including comparisons across stimulus types

Moreover, sublexical factors such as phonological iconicity (sound-to-meaning correspondences) and intrapersonal factors (e.g., subjective mood, anxiety) can influence emotional word processing (e.g., Eden et al.; Sereno et al.; Ullrich et al., in this book). Regarding sublexical factors, these factors may modulate already stimulus-driven early stages of emotional word processing (Ullrich et al. in this book). Furthermore, anxiety may modulate activity in emotion structures such as the amygdala (involved in emotion detection and emotional response selection) in associative word-learning paradigms (Eden et al. in this book), whereas positive mood may change lexical decisions for positive and negative words via a broadening of attention (Sereno et al. in this book). Also, the induced mood state (via positive or negative film clips) may significantly affect syntactic processing of words. Thus, the interaction between emotion and language can go beyond semantic processing levels (see Verhees et al. in this book).

Nevertheless, an early stimulus tagging stage seems obligatory for all types of emotional stimuli (faces, words, pictures). This is also suggested by studies that compared the time course of emotional picture, emotional face and emotional word processing. These studies suggest that pictures, faces and words do evoke the same electrophysiological signals (e.g., an early posterior negativity component, EPN, as well as a late positive potential, LPP), but the emotion effects elicited at later processing stages may be stimulus-type specific due to a positivity offset elicited by the overall lower arousal levels of words vs. faces and pictures (see Bayer and Schacht; Lüdtke and Jacobs in this book for a discussion of EEG and behavioral results).

## Emotional word processing—current theories and perspectives

What many emotional word processing studies though still leave open is whether the results summarized above are more compatible with traditional associative network models, interactive dual processing models or with an embodied account of word processing. Associative network models of emotions assume that emotional content conveyed by an abstract symbol such as a word or a concrete emotional stimulus such as a picture is rapidly mapped onto conceptual knowledge stored in associative memory networks. The information stored in these networks as nodes includes links to the operations, use, and purpose of the stimulus, as well as its emotional and physiological consequences (e.g., Lang, 1979; Bower, 1981). Importantly, activation of these networks is assumed to partially reactivate the perceptual processing, feeling- and action patterns that occur when directly confronted with an emotion inducing event in real time; an assumption that is also shared by theories of embodied cognition, that view knowledge as grounded in perception and action. Dual processing models (e.g., Paivio, 2010) as well as embodied theories of language processing (e.g., Barsalou et al., 2008) distinguish between two processing systems. Controversy between the two theories exists in the way concrete and abstract stimuli are processed by the two propagated systems (Vigliocco et al., 2009; Kousta et al., 2011; Paivio, 2013, for a discussion). Embodied theories propose a fast linguistic system and a temporally slower imagery-based simulation system (see Ensi-Abassi et al. in this book). Additionally, they assume that experiencing emotions through abstract words is possible only through simulation or reenactment. Theoretically, it has been proposed that on a cortical level, embodied processing of emotional words is laterized to the right hemisphere, whereas a pure linguistic and probably “cold” appraisal of words is more strongly associated with left-hemisphere activation (see Ensi-Abassi et al. Moritz-Gasser et al. in this book).

## Going beyond single words—the impact of self-reference, social relevance and communicative context on emotional word and sentence processing

Compelling evidence that emotional content conveyed by abstract symbols such as words can elicit consciously retrievable affective feeling states comes from recent studies that extended emotion word processing to the domains of social cognition. Going beyond single words, a number of these studies use sentences that differ in self-reference (see Fields and Kuperberg, in this book). Other studies use compound stimuli consisting of pronoun- and article-noun pairs making a reference to the reader's own emotions (e.g., “my fear,” “my pleasure”) or to the emotion of another person (“my fear,” “my pleasure”) or that contain no particular personal reference (see Weis and Herbert, in this book). Some studies are using more complex designs in which participants read emotional trait adjectives in anticipation of an evaluation by a significant communicative sender (see Schindler et al. in this book). Generally speaking, these studies allow a detailed analysis of where and when in the processing stream emotional meaning is discriminated from neutral meaning as a function of the communicative context and the stimuli's personal or social reference (self, other, no reference). Crucially, one particular observation of these studies is that self-reference impacts emotional word processing during later stages of cortical processing, i.e., after an in-depth semantic analysis (N400, LPP) (see Fields and Kuperberg in this book; see also Herbert et al., 2011a,b). Moreover, the self-reference of an emotional word seems to selectively enhance activity in cortical midline structures, possibly generating an awareness, feeling or evaluation that this stimulus and its content refer to one's own emotion (see Herbert et al., 2011c). Nevertheless, the evaluation of self-related emotional words in reference to one's own feelings may not be accompanied by stronger emotional expressive behavior or by stronger physiological changes in heart rate or skin conductance: instead, it appears that appraising other-related emotional words (e.g., “his happiness”) in reference to one's own feelings elicits significant changes in facial muscle activity (see Weis and Herbert, in this book).

Taken together, the results of the studies presented in Chapter 3 argue in favor of a differentiated view of embodied emotional word processing. The studies suggest that the social relevance of the emotional words needs to be taken into consideration. Interestingly, anticipating the evaluation by a communicative partner seems to be sufficient to increase the relevance of an emotional word. This seems to facilitate already early cortical processing in the EPN time window (see Schindler et al. in this book). Moreover, recent studies have extended emotional word processing to the domain of verbal fear learning and to symbolic generalization (see Bennet et al. in this book) and to grammatical aspects in political speech (see Havas and Chapp, in this book) and to the general affective meaning of a word in poetic texts (see Ullrich et al. in this book).

## Where are the words in emotions? affect labeling, emotional language acquisition, multilingualisms and poetic aesthetics

Although the results reviewed above clearly support the notion that words can elicit emotions, yet, there is another line of research showing that language processing can also regulate and change emotion perception of non-verbal emotional signals (e.g., Lieberman et al., 2011; Herbert et al., 2013; see Lindquist et al. in this book for discussion). Viewed from a developmental perspective of the human brain, emotion processing may be significantly influenced by language as soon as children learn to use words and verbal labels for emotion expression and emotion categorization (see Lindquist et al. in this book). This implies that in the adult brain, language and emotions are inextricably intertwined, influencing each other on different levels of cerebral, peripheral, subjective and behavioral responding. Due to this bidirectional link between emotion and language, experimental approaches probing learning of new emotion concepts in adults in different languages as well as approaches investigating emotion processing in mono- vs. bilinguals or multilingual speakers seem to be especially fruitful to better understand this interaction (e.g., see Caldwell-Harris; Ferré et al. in this book).

## Conclusion

As outlined above, the articles included in this book *The Janus Face of Language: Where are the Emotions in Words and Where are the Words in Emotions?* can provide a conclusive theoretical and empirical answer to the questions raised by the Topic Editors Herbert, Ethofer, Fallgatter, Walla, and Northoff. The authors of the in total 24 articles theoretically and empirically illuminate the key aspects of the relationship between language and emotion. They provide answers to how information about an emotion is decoded from abstract stimuli such as words, and how the emotional content of a word is processed in the brain. They furthermore highlight the role bodily physiological changes and self- and socially relevant contexts play in the processing and generation of emotional word meaning.

## Summary and structure of the chapters

The articles are grouped into four chapters: **Chapter 1** comprises articles with a strong theoretical focus. These articles discuss recent theoretical views that exist in explaining the emotion-language link with regard to written language. In addition, empirical research focusing on word corpora analyses is included in Chapter 1 investigating the major core affective dimensions underlying the appraisal of emotional words in different languages. **Chapter 2** comprises several experimental studies investigating the brain structures and the time course of emotional word processing. These studies also lay special focus on the effects of task-, sublexical, and intrapersonal factors. Moreover, they shed light on the questions of how affective core dimensions (e.g., emotional valence, emotional arousal or affective origin) influence emotion word processing, the interaction between words and the direction of behavior (approach vs. withdrawal). The studies summarized in **Chapter 3** extend emotional word processing to the domains of social cognition. They provide evidence that the interaction between words and emotions must also be seen in a broader context that takes intrapersonal (self-reference), social factors (sender-receiver characteristics) and the sender's communicative intentions into consideration. Finally, the studies summarized in **Chapter 4** extend the research on emotional word processing to the domains of aesthetics and poetic text, bi- and multilingualism, i.e., areas of psycholinguistic and psychological language research that have developed only recently.

## Author contributions

All authors listed have made a substantial intellectual contribution to the work, and approved it for publication.

### Conflict of interest statement

The authors declare that the research was conducted in the absence of any commercial or financial relationships that could be construed as a potential conflict of interest.
